# Inhibition of poly (ADP-Ribose) polymerase-1 in telomerase deficient mouse embryonic fibroblasts increases arsenite-induced genome instability

**DOI:** 10.1186/2041-9414-1-5

**Published:** 2010-05-26

**Authors:** Resham L Gurung, Lakshmidevi Balakrishnan, Rabindra N Bhattacharjee, Jayapal Manikandan, Srividya Swaminathan, M Prakash Hande

**Affiliations:** 1 Department of Physiology, Yong Loo Lin School of Medicine, National University of Singapore, Singapore 117597; 2 Department of Anatomy and Cell Biology, University of Western Ontario, London, ON N6A 5C1, Canada

## Abstract

**Background:**

The telomerase enzyme is a viable target for anti-cancer therapy given the innate differences in telomerase activity between tumour cells and normal somatic cells. However, the time lag between telomerase inhibition and telomeres becoming critically short to trigger cell death, allows cancer cells to acquire drug resistance. Inhibition of DNA repair pathways along with telomerase could be an alternative strategy to enhance anti-tumour effects and circumvent the possibility of drug resistance. Poly (ADP-Ribose) Polymerase-1 (PARP-1), an important DNA damage sensor and a DNA repair factor, has important roles in maintaining telomeres and chromosomal stability. In this study, the effects of combined inhibition of PARP-1 and telomerase in mouse embryonic fibroblasts (MEFs) following sodium arsenite exposure (a carcinogen and potent DNA damaging agent), were evaluated.

**Results:**

Inhibition of PARP in telomerase deficient MEFs induced an increase in arsenite-induced DNA damage as compared to control cells. Combined inhibition also resulted in enhanced genomic instability, demonstrated by elevated micronuclei induction and chromosomal aberrations with decreased cell survival. In addition, telomerase inhibition in PARP-1 deficient MEFs led to greater telomere shortening and increased genomic instability.

**Conclusions:**

Our study demonstrated that the co-inhibition of PARP-1 and telomerase in MEFs rendered cells more susceptible to DNA damaging agents. Hence, these results offer support for the use of combined inhibition of PARP-1 and telomerase as a strategy to minimise the problems associated with long-term telomerase inhibition in cancer therapeutics.

## Background

Telomeres are specialised dynamic structures at the ends of linear eukaryotic chromosomes consisting of non-coding DNA repeats (TTAGGG)_n _and associated proteins [1,2]. These terminal DNA-protein complexes function as protective caps preventing chromosomal end-to-end fusions and the recognition of chromosomal ends as damaged DNA [3]. Telomeres shorten with each cell division, eventually triggering senescence [4,5]. In contrast, majority of tumour cells overcome telomere-mediated senescence via the activation of telomerase enzyme [6].

Telomerase contains two core components, an RNA subunit (hTERC and mTERC in human and mouse respectively), which provides the template for replenishment of telomeres [7] and a catalytic protein subunit, telomerase reverse transcriptase (hTERT or mTERT) that adds telomeric repeats to existing telomeres [8]. Deletion of mTERC in mice resulted in the shortening of telomeres leading to increased genomic instability and reduction in growth rate [9-11]. In addition, these studies have also demonstrated that no phenotypic differences occur in the first generation mice lacking mTERC component. The abrogation of telomerase results in the reduction in cell proliferation only after telomeres are critically short.

Numerous DNA repair proteins along with the telomerase complex have been shown to have pivotal roles in the maintenance of telomere homoeostasis without any effect on telomerase activity. Dysfunctional telomeres, resulting from the loss of telomeric repeats or the loss of function of telomere-associated proteins, trigger DNA damage responses similar to that observed for DNA breaks [12-14].

Poly (ADP-ribose) polymerase-1 (PARP-1), a member of the PARP family, is a DNA damage sensor [15,16] that allows for DNA repair upon binding to DNA strand breaks. This is effected by the post-translation mediation of downstream proteins in the base excision repair pathway [17-19]. Poly (ADP-ribose) polymerases (PARPs) mediates addition of ribose moiety using NAD^+ ^as substrate in post translational modification of histones and other nuclear proteins that contributes to the survival of cells following DNA damage[20,21]. PARP-1 also plays a role in telomere maintenance [22,23]. PARP-1^-/- ^mouse embryonic fibroblasts (MEFs) exhibited heightened genomic instability and telomere dysfunction following exposure to DNA damaging agents [24,25]. Recently, it was shown that in the absence of telomere dysfunction, PARP-1 appears sporadically at telomeres but following DNA damage, PARP-1 localised to the damaged telomeres through its interaction with the telomere repeat binding factor 2, TRF2 [26].

Apart from the complex protein network involved in telomere homeostasis, telomerase enzyme plays a dominant role in telomere maintenance in tumour cells. Telomerase inhibition has become an attractive target for cancer therapeutics due to specific targeting of tumour cells [27-29]. However, a potential drawback of telomerase inhibition as a chemotherapeutic agent is that telomeres must become critically short before cytotoxic effects are observed in cancer cells. This lag phase may allow cancer cells to adapt using mechanisms such as alternative lengthening of telomeres, to counteract telomere shortening triggered by the absence of telomerase.

We recently reported that cells with dysfunctional telomeres are susceptible to DNA damage induced by sodium arsenite [30]. Although this study highlights the protective role of telomeres in the event of DNA damage, it also draws attention to the requirement of critically short telomeres for the onset of cytotoxicity. Interestingly, microarray analysis showed differential expression of PARP-1 in wild type and G1-mTERC^-/- ^MEFs [30]. Thus, we wanted to test if the inhibition of PARP-1 in mTERC^-/- ^MEFs could sensitise cells to DNA damaging agents and whether this combinatorial approach can be utilised to sensitise cells to chemotherapeutic agents.

## Results

### PARP inhibition in MEFs lacking telomerase RNA component (mTERC^-/-^) induced elevated arsenite induced DNA damage

In our recent study, we observed an up-regulation of PARP-1 expression following arsenite treatment, which was higher in MEFs deficient in TERC component as compared to wild type [30]. We sought to evaluate whether the inhibition of PARP sensitises mTERC^-/- ^MEFs to sodium arsenite-induced DNA-damage. Wild type and mTERC^-/- ^MEFs, pre-incubated with 3-aminobenzoamide (3-AB; A competitive inhibitor of PARP) [31,32], were treated with different doses of sodium arsenite (As^3+^). Single cell gel electrophoresis assay under alkaline conditions, which allows for the analysis of all types of DNA damage, including double strand breaks, single strand breaks, and alkali labile sites, was carried out to estimate the extent of DNA damage induced by arsenite treatment.

**Figure 1:**
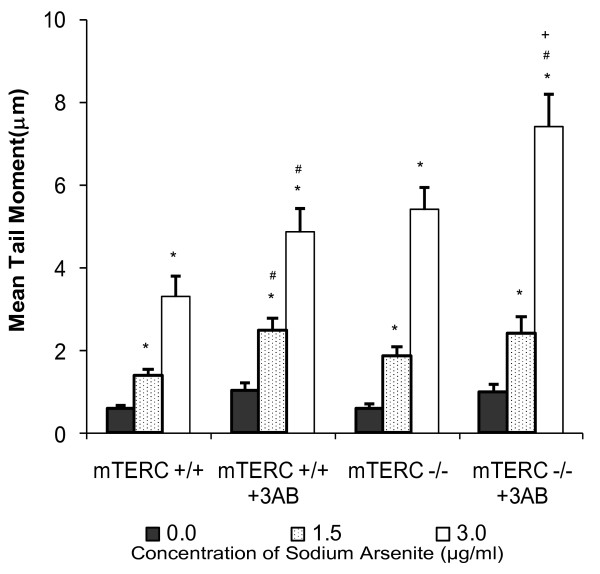
**DNA damage as measured by the comet assay in different cell types**. Comet assay was used to assess the extent of DNA damage in terms of tail moment (fraction of DNA in the tail). mTERC^+/+ ^and mTERC^-/- ^MEFs were treated with arsenite [Untreated (*Black columns*), 1.5 μg/ml (11.5 μM) (*dotted columns*) and 3.0 μg/ml (23 μM) (*white columns*)] in the presence or absence of 3-AB. PARP inhibition in mTERC^-/- ^MEFs led to greater increase in DNA damage as compared to wild type MEFs. Data is represented as mean ± SE from three independent experiments. *p < 0.05 when treated cells are compared to respective untreated controls. ^#^p < 0.05 when 3-AB treated cells are compared to respective 3-AB untreated cells in the presence or absence of arsenite. ^+^p < 0.05 when 3-AB treated mTERC^-/- ^cells are compared to 3-AB treated mTERC^+/+ ^in the absence or presence of arsenite treatment.

Arsenite treatment resulted in elevated DNA damage in mTERC^-/-^, 3-AB treated mTERC^-/- ^and wild type MEFs compared to untreated wild type MEFs (Fig. 1). DNA damage following 24 hours of As^3+ ^treatment was greater in mTERC^-/- ^compared to wild type MEFs. Of all the treatments, the greatest extent of DNA damage was observed in 3-AB exposed mTERC^-/- ^MEFs. At 3.0 μg/ml of As^3+^, mTERC^-/- ^MEFs exhibited significantly higher levels of DNA damage compared to wild type cells. This difference was enhanced following PARP inhibition (Fig. 1), suggesting that the inhibition of PARP in cells with dysfunctional telomeres renders higher sensitivity to As^3+^-induced DNA damage.

### Inhibition of PARP in mTERC^-/- ^MEFs resulted in elevated arsenite-induced chromosomal instability

**Figure 2:**
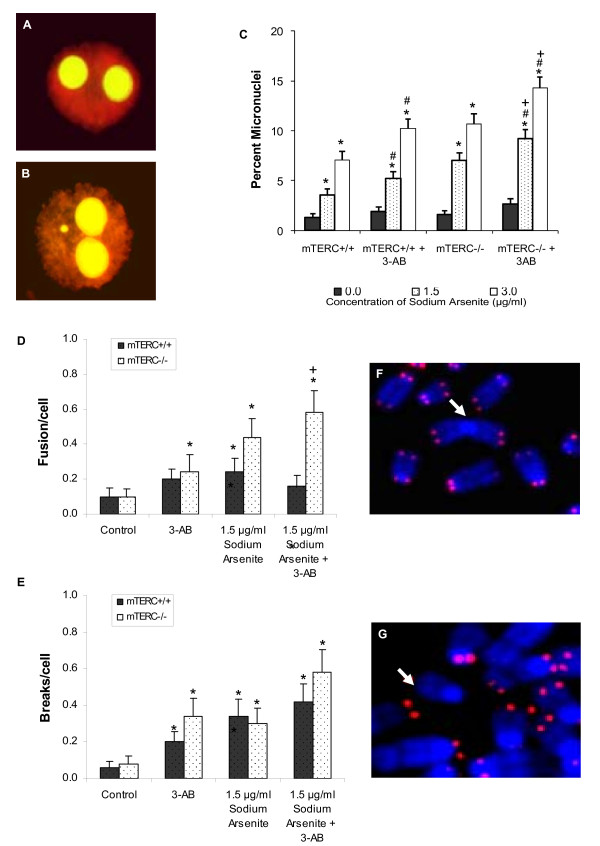
**Effects of PARP inhibition in mTERC^+/+ ^and mTERC^-/- ^MEFs on chromosome stability**. (A) Acridine orange stained binucleated cell with no MN, indicating no apparent damage. (B) Acridine orange stained binucleated cell with MN from PARP inhibited mTERC^-/- ^MEFs following 24 hour arsenite treatment. (C) Histogram of micronuclei formation, in mTERC^+/+ ^MEFs and mTERC^-/- ^MEFs treated with sodium arsenite [Untreated (*Black columns*), 1.5 μg/ml (11.5 μM) (*dotted columns*) and 3.0 μg/ml (23 μM) (*white columns*)] in the presence or absence of 3-AB. mTERC^-/- ^MEFs displayed a greater extent of MN formation as compared to wild type MEFs at each concentration of arsenite and inhibition of PARP-1 in both MEFs further increased this difference. Frequency of chromosomal aberration such as fusion (D) and breaks (E) per cell as analysed by PNA FISH in mTERC^+/+ ^MEFs (*black columns*) and in mTERC^-/- ^MEFs (*white columns*) following treatment with 3-AB and/or sodium arsenite [1.5 μg/ml (11.5 μM)]. Majority of chromosomal breaks and fusions were observed in PARP inhibited mTERC^-/- ^MEFs. Representative images of (F) fusion (arrow) and (G) breaks (arrow) from PARP inhibited mTERC^-/- ^MEFs following 24 hour treatment with arsenite. Data is represented as mean ± SE from three independent experiments. *p < 0.05 when treated cells are compared to respective untreated controls. ^#^p < 0.05 when 3-AB treated cells are compared to respective 3-AB untreated cells in the presence or absence of arsenite. ^+^p < 0.05 when 3-AB treated mTERC^-/- ^cells are compared to 3-AB treated mTERC^+/+ ^in the absence or presence of arsenite treatment.

We have demonstrated in previous studies that arsenite exposure resulted in genomic instability in PARP-1^-/- ^and mTERC^-/- ^MEFs [25,30]. We hypothesised that the inhibition of PARP-1 in telomerase-deficient cells may lead to enhanced arsenite-induced chromosomal instability. To examine this possibility, micronuclei (MN) analysis, a reliable indicator of chromosomal damage and genomic instability, was used. Consistent with previous reports, following 24 hours of arsenite treatment, there was a dose dependent increase in the percentage of MN in wild type and mTERC^-/- ^MEFs, with mTERC^-/- ^MEFs showing greater incidences of MN formation compared to wild type cells (Fig. 2C). Following the chemical inhibition of PARP in these MEFs, there was an increase in MN formation in both the cell lines compared to untreated control cells. mTERC^-/- ^MEFs treated with 3-AB displayed significantly increased percentage of MN compared to 3-AB treated wild type MEFs at each dose of arsenite (Fig. 2C).

The presence of MN is a result of the exclusion of chromosomes or chromosomal fragments from the daughter nuclei. Consequently, we undertook fluorescence in situ hybridisation (FISH) analysis using telomeric PNA probes to analyse structural chromosome aberrations, particularly end to end fusions and chromosomal breaks. Chromosome preparations were carried out only for the 1.5 μg/ml dose of arsenite as it was not possible to obtain metaphases at the higher dose of arsenite. 3-AB treated mTERC^-/- ^MEFs displayed greatest incidences of chromosomal fusions and chromosomal breaks following As^3+ ^treatment (Fig. 2D and 2E). Moreover, the highest percentages of aberrant cells were also observed in PARP inhibited mTERC^-/- ^MEFs following arsenite treatment (56%).

### PARP-1 inhibition sensitised telomerase deficient mouse cells to arsenite induced cell death

**Figure 3:**
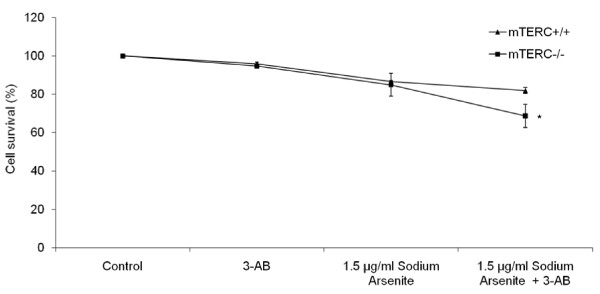
**Inhibition of PARP in mTERC^-/- ^led to greatest decrease in cell survival**. Cell viability was analysed by crystal violet assay in mTERC^+/+ ^and mTERC^-/- ^following 24 hours of arsenite treatment, with and without 3-AB. Following exposure to arsenite, 3-AB treated mTERC^-/- ^cells showed significant decrease in cell survival as compared to 3-AB treated mTERC^+/+ ^cells (*p < 0.05). Data is represented as mean ± SE from three independent experiments.

To investigate the effect of PARP-1 inhibition in mTERC^-/- ^MEFs on cellular proliferation, we examined cell viability using crystal violet assay. Following PARP inhibition with 3-AB, the reduction in cell viability was minimal in wild type and mTERC^-/- ^MEFs. However, upon addition of 1.5 μg/ml arsenite, 19% decrease in cell survival was observed in 3-AB treated wild type MEFs while 3-AB treated mTERC ^-/- ^MEFs displayed an even greater decrease (32%) in cell viability (Fig. 3). This indicated that the combined inhibition of PARP-1 and telomerase increased the sensitivity of MEFs to arsenite-induced genomic instability and reduced viability.

### Combined inhibition of telomerase and PARP reduced telomere length and increased genomic instability in mouse cells

**Figure 4:**
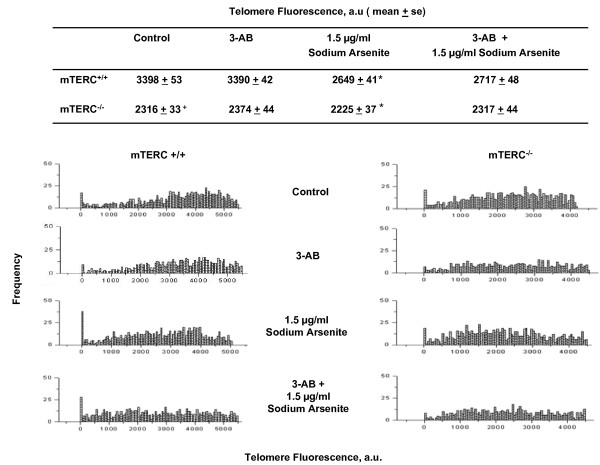
**Telomere length measurements by Q-FISH in different cell types used**. Frequency distributions of telomere fluorescence in mTERC^+/+ ^and mTERC^-/- ^MEFs from Q-FISH studies on metaphases prepared from surviving cells following treatment with PARP inhibitor and arsenite. Average telomere fluorescence intensity was significantly lower (^+^p < 0.05) in untreated mTERC^-/- ^as compared to untreated mTERC^+/+ ^cells. Arsenite treatment led to significant decrease (*p < 0.05) in average mean telomere florescence intensity in both mTERC^+/+ ^cells and mTERC^-/- ^cell as compared to respective untreated controls. Changes in distribution of frequency of cells with shorter telomere fluorescence intensity observed following treatment with 3-AB and arsenite in both mTERC^+/+ ^and mTERC^-/- ^cells.

Previous reports have shown that decreased level of PARP-1 activity either with pharmacological inhibitor or *PARP-1 *deletion in mammalian cells resulted in shorter telomere lengths compared to normal cells without any changes in telomerase activity [22,23]. The effect of PARP inhibition and arsenite treatment on telomere lengths was investigated by quantitative FISH (Q-FISH) in mTERC^-/- ^MEFs. 3-AB exposure for 48 hours did not induce significant changes in telomere length. However, extensive telomere shortening was observed following exposure to arsenite in wild type MEFs (Fig. 4). Interestingly, the frequency of chromosomes with shorter telomere length following arsenite treatment was highest in PARP inhibited MEFs. PARP inhibited mTERC^-/- ^MEFs also displayed increased chromosomal fusions with higher degree of telomere shortening compared to untreated controls.

**Figure 5:**
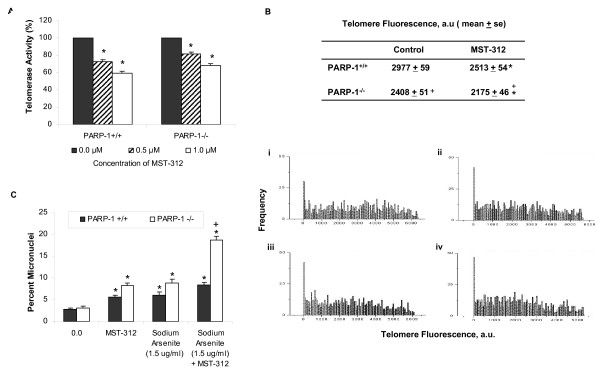
**Telomerase inhibition in PARP-1 deficient MEFs reduced telomere length and increased genomic instability. **(A) Measurement of telomerase activity in PARP-1^+/+ ^and PARP-1^-/- ^MEFs with untreated controls (*black columns*), 0.5 μM (*striped columns*) and 1.0 μM (*white columns*) of MST-312 for 72 hours. (B) Telomere length frequency distribution in (i) PARP-1^+/+ ^MEFs (ii) PARP-1^-/- ^MEFs (iii) PARP-1^+/+ ^MEFs with MST-312 and (iv) PARP-1^-/- ^MEFs with MST-312. Telomerase inhibition in PARP-1 deficient MEFs resulted in greatest decrease in telomere length. (C) Histogram of micronuclei formation in PARP-1^+/+ ^MEFs (*black *columns) and PARP-1^-/- ^MEFs (white columns) with sodium arsenite and MST-312 treatment. MN frequency was significantly increased in telomerase inhibited PARP-1 deficient MEFs following exposure to 1.5 μg/μl of sodium arsenite. Error bars indicate standard error between three independent experiments. *p < 0.05 compared with respective untreated controls. ^+^p < 0.05 compared between MST-312 treated cells with or without arsenite treatment.

Current anti-cancer approaches targeting telomerase are varied, ranging from RNA interference of the RNA or TERT component to identification of novel compounds targeting telomerase activity. We employed MST-312, a synthetic telomerase inhibitor [33] to impair telomere homeostasis in both wild type and PARP-1^-/- ^MEFs. MEFs were treated with 0.5 μM and 1.0 μM of MST-312 for 72 hours and telomerase activity was evaluated using TRAP assay. As shown in Fig 5A, telomerase activity was kept at a significantly reduced level in both wild type and PARP-1^-/- ^MEFs with 72 hours of MST-312 treatment. We then investigated the effect of MST-312 treatment on telomere lengths in MEFs by Q-FISH following 15 days of 1.0 μM MST-312 treatment. The basal telomere length in PARP-1^-/- ^MEFs was lower compared to the wild type MEFs, consistent with previous reports [22,23]. More importantly, MEFs exposed to dual inhibition of telomerase and PARP-1 activity exhibited shortest telomere length (Fig. 5B). The incidence of micronuclei formation following arsenite exposure, also doubled in MEFs with both PARP-1 and telomerase activity inhibited as compared to MEFs with only PARP or telomerase inhibited (Fig. 5C). These findings thus demonstrate that the inhibition of telomerase activity with MST-312 in PARP-1^-/- ^MEFs led to accelerated telomere shortening, rendering these cells more susceptible to arsenite-induced genomic instability.

### Differential gene expression patterns in PARP-1^-/- ^and mTERC^-/- ^MEFs compared to normal MEFs

**Figure 6:**
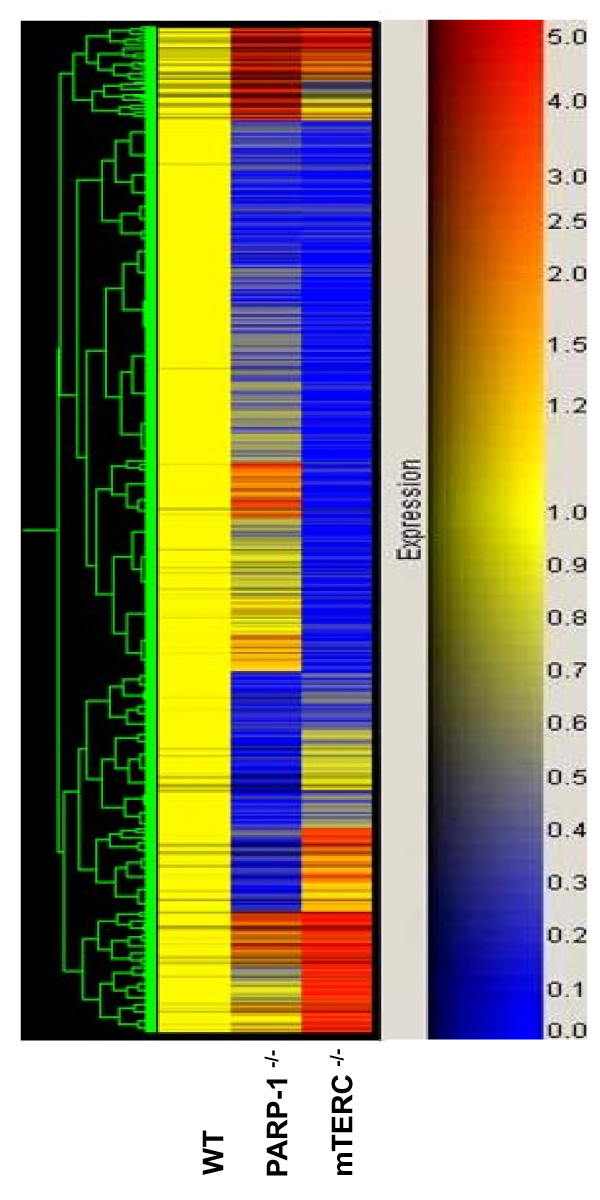
**Gene expression data obtained using Affymetrix Mouse Genome 430 2.0 GeneChip**. 311 differentially expressed probes with < 0.05 and fold change of >2.0 in at least one of the experimental conditions are represented in cluster diagrams. Each column represents a single experiment condition and each row represents a single gene. Expression levels are coloured red for up regulation and blue for down regulation according to the colour scale.

It is well established that the absence of a gene can potentially trigger transcriptional regulation of many compensatory genes. To determine the gene expression profile of PARP-1^-/- ^and mTERC ^-/- ^MEFs compared to that of wild type MEFs, microarray technology was employed. The effect of PARP-1 and mTERC on genes involved in cell proliferation, DNA repair and telomere maintenance was thus examined. As shown in Fig 6, differential gene expression profiles were observed in these MEFs when compared to wild type MEFs. Majority of the genes were either up-regulated or down-regulated in both PARP-1^-/- ^and mTERC ^-/- ^as compared to wild type MEFs (Table 1).

**Table 1:** Functional categorisation of differentially expressed genes in wild type, mTERC ^-/- ^and PARP-1^-/- ^MEFs following arsenite treatment.

Gene Name	GeneBank ID	**mTERC**^**-/-**^	**PARP-1**^**-/-**^
**Cell death**			
Aifm2	AK017403	6.50	4.50
Bcl2l11	BB667581	3.47	2.22
Siva	AF033112	3.37	1.24
Sphk1	AF068749	3.22	3.35
Pik3r1	M60651	1.24	-2.45
Birc1f	AI451585	-1.58	-2.15
IAP1	NM_007464	-2.55	-1.72
FLG	M33760	-3.13	-1.65
Casp9	BB815299	-3.78	1.68
Faim-L	NM_011810	-4.24	-1.38
Fam	NM_007983	-8.56	-1.33
Birc6	BM228823	-9.20	-2.41
Dad1	BI966630	-9.29	-1.67
Cycs	NM_007808	-9.47	-2.83
Stk17b	AI661948	-9.94	1.01
Il-6	NM_031168	-12.48	-8.72
Bip	NM_022310	-20.04	-3.15
			
**DNA damage response**			
Brca2	NM_009765	-1.64	-2.09
Xrn2	NM_011917	-1.82	-1.01
H2afx	NM_010436	-2.04	-2.03
Ddb2	AI131584	-2.07	1.16
Rad51ap1	BC003738	-2.31	-2.59
Ercc5	BM198879	-2.32	-1.33
Gadd45a	NM_007836	-2.57	-1.67
Mlh1	NM_026810	-2.82	-1.46
Msh6	U42190	-2.94	-1.56
Chek2	NM_016681	-3.09	-1.72
1110013J05Rik	NM_025392	-3.17	-2.17
Lig4	AW545311	-4.01	-1.95
Sod1	BC002066	-6.15	-1.81
Mapk14	BC012235	-7.63	-2.63
			
**Telomere homeostasis**			
Smg6	BC006644	3.18	6.26
Adprt1	BB767586	2.49	-4.06
Terf2	BI439979	2.01	-2.23
Hspa1b	M12573	1.28	1.43
Blm	NM_007550	1.02	-2.77
DNA-PK	D87521	-1.98	-3.18

Expression profile of selected genes, which were differentially expressed to a fold change of 2.0 or more in one of the treatments are given. Data indicate the fold increase (positive values) or decrease (negative values) in treated samples compared to corresponding normal controls.

## Discussion

Several lines of evidence indicate that the activation of telomerase and the subsequent stabilisation of the telomeres are vital for the growth for majority of tumour cells. While most tumour cells express telomerase, somatic cells generally do not express this enzyme [6]. Telomerase thus serves as a potential target for cancer therapy. The main drawback is the duration required for telomere length to reach a critical level before triggering senescence and/or apoptosis, which allow tumour cells time to develop resistance to anti-telomerase agents. Mice lacking telomerase RNA component survived but exhibited telomere shortening and increased chromosome instability [9]. Telomerase deficient mouse cells were also viable in culture and found to employ telomerase independent mechanisms to maintain their telomeres [11,34]. We have demonstrated in earlier studies that MEFs with dysfunctional telomeres exhibited sensitivity towards arsenite induced genomic instability treatment [30]. Similar observations were made in the present study whereby mTERC^-/- ^MEFs sustained shorter basal telomere length and exhibited significantly higher arsenite induced genomic instability as compared to mTERC^+/+ ^MEFs. However to counteract the limitations of using telomerase inhibition alone, we explored the effects of a combinatorial approach of targeting telomerase activity and PARP-1 using PARP-1^-/- ^and mTERC^-/- ^MEFs with arsenite exposure.

Pharmacological inhibition of PARP in telomerase null MEFs increased the sensitivity to arsenite-induced cytotoxicity and genotoxicity. The inhibition of Tankyrase 1, a member of PARP family, in human cancer cells, has been shown to enhance telomere shortening and hasten cell death [35]. As observed in this study, the inhibition of PARP-1 activity in telomerase deficient MEFs led to reduced cell survival, which were accompanied by elevated genomic instability. Concurrently, we treated PARP-1^-/- ^MEFs with a synthetic telomerase inhibitor MST-312. Interestingly, the telomere length analysis by Q-FISH indicated that the cells deficient in PARP-1 activity with pharmacological reduction in telomerase activity displayed the shortest average telomere length. Telomerase inhibition in PARP-1^-/- ^MEFs enhanced arsenite-induced genomic instability as well. Mammalian telomeric ends are protected by proteins such as TRF2 which stabilise the loop structure of the telomeres [36,37]. A recent report has shown that the localisation of PARP-1 to damaged telomeric ends is partly attributed to its interaction with the TRF2 protein [26]. Arsenite-induced damage at the chromosome ends may trigger the recruitment of PARP-1 to the telomeres to mediate repair. Thus, the increased sensitivity of telomerase deficient MEFs with PARP-1 inhibition to arsenite damage may be due to the lack of repair at both non-telomeric DNA and telomeric ends. The present study thus demonstrates the potential of combined inhibition of PARP-1 and telomerase for cancer therapy.

Gene expression studies revealed differential gene expression patterns in PARP-1^-/- ^and mTERC^-/- ^compared to wild type MEFs. Genes that are differentially regulated in both the cell types include genes involved in the biological processes such as cell growth and/or maintenance, DNA damage response, repair and telomere maintenance. More importantly, about 42 genes which are important for DNA damage signalling and repair and cell death were shown to be altered in their expression patterns. The gene expression level of BRCA2 was reduced in PARP-1^-/- ^and mTERC^-/- ^compared to wild type MEFs suggesting potential interplay of these genetic factors. It was recently found that the targeting of PARP-1 in cells defective in homologous recombination due to BRCA1 or BRCA2 dysfunction results in chromosomal instability, cell cycle arrest and subsequent apoptosis [38,39]. Some of the PARP inhibitions are also currently used in combination with chemotherapy in clinical trials [40]. Hence, understanding the changes in gene expression profiles may provide potential insights into selecting appropriate candidates for further combinatorial studies.

## Conclusions

Integrating the findings from both models used in this study, it is possible to infer that telomerase inhibition and the consequent telomere shortening sensitises MEFs to DNA damaging agents. Our study demonstrated that the combined inhibition of PARP-1 and telomerase in MEFs rendered cells more susceptible to DNA damaging agents. Hence, these results offer support for the use of co-inhibition of PARP-1 and telomerase as a strategy to overcome the limitations associated with telomerase inhibition alone for cancer therapy.

## Materials and methods

### Cell culture and drug treatment

Wild type, PARP-1^-/- ^and mTERC^-/- ^MEFs (Kindly provided by Dr. Zhao-Qi Wang, Germany and Dr. Han-Woong Lee, South Korea respectively) were cultured in Dulbecco's Modified Eagles Medium (DMEM) supplemented with 10% foetal bovine serum (Hyclone, USA) and 100 U/ml of penicillin/streptomycin (Gibco, USA). To evaluate the response of PARP-1 and telomerase deficiency to arsenite-induced damage, cells in exponential growth phase (at about 70% confluence) were exposed to two different doses of sodium m-arsenite [(As^3+^; Sigma, USA) 1.5 μg/ml (11.5 μM) and 3.0 μg/ml (23 μM)] for 24 hours. PARP-1^-/- ^MEFs were pre-treated with 1 μM telomerase inhibitor, MST-312 for 48 hours and mTERC^-/- ^MEFs were treated with 3 mM 3-Aminobenzamide (3-AB; Sigma, USA) for 24 hours prior to exposure to As^3+^. All cells were maintained in a humidified 5% CO_2 _incubator at 37°C.

### Assay for cell viability

Following drug treatment, cells were washed with phosphate buffered saline (PBS). Crystal violet solution (0.75% crystal violet in 50% ethanol: distilled water with 1.75% formaldehyde and 0.25% NaCl), which stains DNA by binding to nuclear proteins, was added to the culture wells and incubated for 20 minutes at room temperature. Following successive PBS washes to remove excess crystal violet solution, wells were air dried and 1% sodium dodecyl sulphate in PBS was added to lyse the cells and solubilise the dye. Cell viability was measured at 590 nm absorbance and expressed as the percentage of controls.

### Alkaline single cell gel electrophoresis (Comet) assay

Cells were treated with As^3+ ^for 24 hours with the doses mentioned earlier. The treated cells were harvested by trypsinisation and washed in ice-cold PBS. The cells were then suspended in Hank's balanced salt solution (Sigma, USA) and mixed with 0.7% low melting point agarose (at 37°C). The cells were then applied on Comet slides (Trevigen, USA), and subjected to lysis (2.5 M NaCl, 0.1 M pH 8 EDTA, 10 mM Tris base, 1% Triton X) at 4°C for 1 hour. The slides were loaded into a gel electrophoresis tank in 0.3 M NaOH, pH 13 with EDTA, allowed to denature for 40 minutes, and electrophoresis was done as per vendor's suggestions. After electrophoresis, slides were briefly rinsed in neutralization buffer (500 mmol/L Tris-HCl, pH 7.5), air-dried, and stained with SYBR green dye (Trevigen, USA). One hundred randomly selected nuclei were examined per sample using Comet Imager Software (Metasystems, Germany). Extent of DNA damage was expressed as a measure of comet tail moments, which corresponds to the fraction of DNA in the comet tail.

### Cytokinesis-blocked micronucleus analysis (CBMN)

Following arsenite treatment, cells were incubated with 4 μg/ml cytochalasin B (Sigma, USA) for 22 hours and processed as described earlier [41,42]. One thousand binucleated cells for each sample were scored for the presence of MN under a Zeiss Axioplan 2 imaging fluorescence microscope (Carl Zeiss, Germany) with appropriate triple band filter.

### Telomeric Repeat Amplification Protocol (TRAP) Assay

Telomerase activity was measured by the commercially available TRAPeze^® ^XL Telomerase Detection Kit (Chemicon International) according to the manufacturer's instructions.

### Peptide nucleic acid-fluorescence in situ hybridisation (PNA-FISH)

Following arsenite treatment, cells were released from the treatment and allowed to grow for 24 hours in fresh media. Cells were arrested at metaphase with 0.1 μg/ml colcemid (Gibco, USA). The cells were harvested and subjected to hypotonic treatment of 0.03 M sodium citrate buffer at 37°C for 20 minutes followed by fixation in Carnoy's fixative. Fluorescence in situ hybridisation (FISH) was performed using telomere specific PNA probe labelled with Cy3 and the cells were counterstained with 4', 6-Diamidino-2-phenylindole (DAPI, Vectashield) [11]. Fifty metaphases per sample were captured using the Zeiss Axioplan 2 imaging fluorescence microscope and analysed using the in situ imaging software (Metasystems, Germany) for chromosomal aberration analysis. Ten metaphase spreads per sample were analysed for telomere length measurement using the ISIS imaging software (Metasystems, Germany).

### Gene expression analysis

Gene expression profiles were generated for Wild type, PARP-1^-/- ^and mTERC^-/- ^MEFs by microarray gene chip assay. Total RNA was extracted (RNeasy kit, Qiagen, Germany), and double-stranded cDNA was synthesized from 5 μg of total RNA using Superscript system (Invitrogen, USA) primed with T7-(dT)-24 primer. For biotin-labelled cRNA synthesis, *in vitro *transcription reaction was done in the presence of T7 RNA polymerase and biotinylated ribonucleotides (Enzo Diagnostics, USA). The cRNA product was purified (RNeasy kit, Qiagen, Germany), fragmented, and hybridized to Affymetrix GeneChip Mouse Genome 430 2.0 in a Gene chip hybridization oven 640 (Affymetrix Inc., USA) as per the Gene Chip Expression Analysis manual (Affymetrix Inc., USA). After 16 hours of hybridisation, the gene chips were washed and stained using the Affymetrix Fluidic station and scanned by Gene Array Scanner (Affymetrix Inc., USA). Image data were normalized and statistically analysed using Gene Spring 7.2 (Silicon Genetics, USA). Microarray experiments were repeated twice in order to confirm the differential gene expression. More than 300 genes with *P *< 0.05 (one-way ANOVA) were differentially expressed and they were annotated according to GO-biological process. Subsequent data analysis involved Agglomerative average-linkage hierarchical clustering for finding different patterns and levels of gene expression.

### Statistical analysis

Statistical significance in the data sets was assessed using Student's t-test using Microsoft Excel 2003 (Microsoft Corp., USA) and two-way ANOVA, using Graphpad Prism. The difference was considered to be statistically significant when the p values are < 0.05.

## Competing interests

The authors declare that they have no competing interests.

## Authors' contributions

RLG participated in the design of experiments, carried out the experiments, analysed the data and drafted the manuscript. LD was involved experimental design, chromosomal analysis, and manuscript preparation. RNB carried out the microarray experiments and JM undertook the bioinformatics analysis. SS was involved in experimental design and manuscript preparation. MPH oversaw the overall experimental design and coordinated all research work and manuscript preparation. All authors read and approved the final manuscript.
